# Antimicrobial Activity and Chemical Composition of “Kpètè-Kpètè”: A Starter of Benin Traditional Beer Tchoukoutou

**DOI:** 10.1155/2017/6582038

**Published:** 2017-03-06

**Authors:** Christine N'tcha, Haziz Sina, Adéchola Pierre Polycarpe Kayodé, Joachim D. Gbenou, Lamine Baba-Moussa

**Affiliations:** ^1^Laboratoire de Biologie et de Typage Moléculaire en Microbiologie, Université d'Abomey-Calavi, 05 BP 1604 Cotonou, Benin; ^2^Laboratoire de Valorisation et de Gestion de la Qualité de Bioingrédient Alimentaire, Université d'Abomey-Calavi, 01 BP 526 Cotonou, Benin; ^3^Laboratoire de Pharmacognosie et des Huiles Essentielles, FSS, Université d'Abomey-Calavi, 01 BP 4521 Cotonou, Benin

## Abstract

The aim of this study was to investigate the antibacterial effect of the crude starter “*kpètè-kpètè*” and lactic acid bacteria used during the production of “tchoukoutou.” To achieve this, a total of 11 lactic acid bacteria and 40 starter samples were collected from four communes. The samples were tested on 29 gram + and − strains by disk diffusion method. The minimum inhibitory and bactericidal concentrations of starter and lactic acid bacteria were determined by conventional methods. Organic acids, sugar, and volatile compounds were determined using the HPLC method. The “kpètè-kpètè” displays a high antibacterial activity against the tested strains. The most sensitive strain was* S. epidermidis* (12.5 mm) whereas the resistance strain was* Proteus mirabilis* (8 mm). All the tested ferment has not any inhibitory effect on* Enterococcus faecalis*. The lactic acid bacteria isolates of Parakou showed the highest (17.48 mm) antibacterial activity whereas the smallest diameter was obtained with the ferment collected from Boukoumbé (9.80 mm). The starters' chemical screening revealed the presence of tannins, anthocyanin flavonoids, triterpenes, steroids, reducing compounds, and mucilage O-glycosides. These compounds are probably the source of recorded inhibition effect. The lactic acid bacteria of the “kpètè-kpètè” could be used to develop a food ingredient with probiotic property.

## 1. Introduction

Major scientific advances have been done in several areas of health sciences for many years mainly in the development of antimicrobial agents. Moreover, pathogenic species such as* Escherichia coli*,* Salmonella *spp.,* Vibrio anguillarum*,* V. alginolyticus, Aeromonas hydrophila*,* A. salmonicida, *and* Yersinia ruckeri* were isolated from gastrointestinal tract (stomach, jejunum, and caecum) samples of poultry, rabbit, fish, and pork [1]. However, humans and animals deaths due to microbial infections (acute respiratory infections, hospital acquired, diarrhea, cancer, and malaria) are reported [2]. One of the main reasons of this situation can be the increasing antibiotic resistance observed over decades. Indeed, the appearance of antibacterial resistance considerably reduced the efficacy of infections treatments. So, pharmaceutical industries find difficult to follow the resistance rate and then develop new effective antimicrobial agents against resistant pathogens [3]. Thus, many multiresistant bacteria are reported in food and clinical samples collected in Benin [4], Côte d'Ivoire [5–8], and other countries in sub-Saharan Africa [9].

To face this public health problem, it is necessary to investigate new method that can help to strengthen conventional antibiotics through the investigation of new antimicrobial molecules. Thus, several paths can be explored including indigenous knowledge through traditional medicine. In spite of its lack of scientific certification, the traditional medicine is known to be efficient to cure several diseases [10]. Among the traditional knowledge, the use of fermented beverage (particularly their lactic bacteria content) to overcome some bacterial infections [11, 12] and food conservation [13] is current. So, the use of lactic acid bacteria could be considered as an alternative to control resistant pathogenic bacterial strains.

Several studies shows that some lactic acid bacteria such as* Lactobacillus *isolated from fermented foods have antimicrobial activity [14, 15]. Up to date the principal microorganisms reported to have probiotic properties are bacteria (*Lactobacillus*,* Bifidobacterium*,* Propionibacterium*,* Enterococcus, *etc.) and yeast (*Saccharomyces*,* Candida, *etc.) [16]. These microorganisms are even used for the formulation of new probiotic products [17].

In Benin, the starter of the most popular traditional sorghum beer named “tchoukoutou” is reported to be used to cure several infections such as stomach pains and diarrhea [12]. However, a part of the preliminary study conducted in 2012 by Kayodé et al. [18] showed that there is no scientific evidence to support the traditional use of the tchoukoutou's starter named “kpètè-kpètè” in infection treatments. Thus, the aim of the present study was to investigate the antimicrobial activity of starter “kpètè-kpètè” and it isolated lactic acid bacteria not only on some reference microorganisms' strains but also on some food and clinical isolated pathogenic bacteria strains.

## 2. Material and Methods

### 2.1. *Kpètè-Kpètè* Sample Collection

The starters used for the study were the ones used in the fermentation of the traditional beer “tchoukoutou” and locally called “kpètè-kpètè.” Samples were collected in four communes selected from the two departments (Atacora and Borgou) displaying the highest production and consumption level of the “tchoukoutou.” The 4 communes are Natitingou (10°18′24′′N; 1°22′30′′E), Boukoumbé (10°18′24′′N; 1°22′30′′E), Tanguiéta (10°37′11′′N, 1°15′52′′E), the department of Atacora (North-West Benin) and Parakou (9°21′00′′N; 2°37′00′′E) in the department of Borgou (Northern-East). In each commune, 10 samples of tchoukoutou's starter were collected for laboratory analysis. Thus, a total of 40 samples were collected and tested for this study.

### 2.2. Tested Strains

The stains used for the antimicrobial test were composed of 12 references strains (*Staphylococcus aureus* ATCC 29213,* S. epidermidis* T22695,* Proteus mirabilis *A24974,* P. vulgaris* A25015,* Micrococcus luteus*,* Streptococcus oralis*,* Enterococcus faecalis* ATCC 29212,* Escherichia coli *ATCC 25922,* E. coli *O157: H7 ATTC 700728,* Salmonella typhi *R 30951401*, Candida albicans* MHMR, and* Pseudomonas aeruginosa* ATCC 27853), 15 clinical and food isolated methicillin resistance* S. aureus *(MRSA), 2 extended-spectrum beta-lactamase* E. coli *strains, and 11 lactic acid bacteria isolated previously from “tchoukoutou” [12].

### 2.3. In Vitro Determination of “Kpètè-Kpètè” Antimicrobial Activity

The antimicrobial activity of the collected starters was evaluated by disk diffusion method [19]. Briefly, 100 *μ*L of 10^6^ CFU/mL microbial suspension was used to inoculate Mueller-Hinton agar by flooding (Bio-Rad, France) [20]. Sterile paper discs impregnated with 30 *μ*L of starters' supernatant (20 mg/mL) were then deposited on the medium. For each test, the experiment is duplicated and a negative control was performed using sterile distilled water. Plates were then left for 15–30 min at room temperature before being incubated at 37°C for 24 to 48 h. Diameters of inhibition zones were then measured using a sliding graduated scale after incubation times (24 h and 48 h).

### 2.4. Antibacterial Activity of Lactic Acid Bacteria Strains

#### 2.4.1. Bacteriocins Extraction

The method describe by Savadogo et al. [21] was used to extract the bacteriocin produced by the lactic acid bacteria. Briefly, each lactic acid bacteria strains were previously mixed in 1000 mL of MRS broth (pH 7.0). For the bacteriocin extraction, a cell-free solution was obtained by centrifuging (10.000 rpm for 20 min, at 4°C) the culture. Thus, the obtained cell-free solution was precipitated with ammonium sulphate (40% saturation) for 2 h at 4°C. Cells were harvested by centrifugation at 20.000 rpm for an hour at 4°C and resuspended in 0.05 M potassium phosphate buffer (25 mL, pH 7.0) for further disk diffusion assay.

#### 2.4.2. Determination of Bacteriocin Activity

The bacteriocin activity was determined by disk diffusion assay [22]. For this assay, the aliquot of each bacteriocin (50 *μ*L) was used to impregnate disk. Once impregnated, the disks were lodged on Mueller-Hinton agar dishes containing 5 · 10^5^ CFU/mL of tested pathogenic strains (*E. coli* O157: H7 ATCC 700728,* Salmonella typhi *R 30951401,* E. coli*,* S. aureus *ATCC 29213, and 2 clinical isolated MRSA). The dishes were then incubated at 30°C or 37°C for 24 h. Diameters of inhibition zones were then measured using a sliding graduated scale [23].

### 2.5. Sensitivity of Antagonistic Substances of Lactic Acid Bacteria to Enzymes

The sensitivity of antimicrobial substances to enzymes was tested according to the method previously described by Savadogo et al. [21]. Briefly, the cell-free supernatants of 13 lactic acid bacteria isolates that showed antimicrobial activity against reference microorganism were collected after centrifugation (7500 ×g for 10 min at 4°C). The pH of the supernatants was adjusted to 6 with 10 N NaOH and treated with 0.2 mg/mL of specific enzymes (Sigma-Aldrich Brasil, São Paulo, Brazil). The used enzymes were lipase (8.6 U/mg in 0.05 M Tris hydrochloride pH 8.0 and 0.01 M CaCl_2_)_,_ chymotrypsin (47 U/mg in 0.05 M Tris hydrochloride pH 8.0 and 0.01 M CaCl_2_)_,_ type x trypsin (15000 U/mg in 0.2 M citrate pH 6.0), pepsin (3.2 U/mL in 0.2 M citrate pH 6.0), and catalase (2.0 U/mg in 10 mM potassium phosphate pH 7.0). The samples of bacteriocins (500 *μ*L) were incubated with the appropriate enzymes (500 *μ*L) for 60 min at 37°C or 25°C (for trypsin, chymotrypsin and catalase). The negative controls contain supernatant solutions and 0.1 M sodium phosphate buffer without enzymes.

### 2.6. Determination of the Starters' Minimum Inhibitory Concentrations (MIC)

The minimum inhibitory concentrations of starter were determined by macrodilution method [24]. Concentrations tested were 10 mg/mL, 5 mg/mL, 2.5 mg/mL, 1.25 mg/mL, 0.625 mg/mL, 0.3125 mg/mL, 0.15625 mg/mL, 0.07812 mg/mL, and 0.03906 mg/mL. Culture medium without starters and without microorganisms was used as controls. Thus, after gently mix, tubes were incubated at 37°C for 18–24 h and growth was indicated by turbidity. The lowest concentration of the starter at which the tested microorganism does not demonstrate visible growth was considered as minimum inhibitory concentrations.

### 2.7. Determination of Lactic Acid Bacteria's Minimum Inhibitory Concentrations (MIC) against* E. coli* ATCC25922

For the determination of the MIC, the crude supernatant obtained after centrifugation (12000*g* for 10 min) of the lactic acid bacteria precultures was consecutively 2-ratio factor diluted up to 512. Antibacterial activities were subsequently carried out with the different concentration of extract of bacteriocin. Thus the lowest dilution which showed a positive antibacterial activity against the tested reference strain was considered as the minimum inhibitory concentration.

### 2.8. Determination of the Starters' Minimum Bactericidal Concentration (MBC)

The minimum bactericidal concentration of the tested microorganisms was determined by subculturing method. Thus, the content of each test tube used in the minimum inhibitory concentration assay that did not show microorganism growth after incubation was streaked on a solid nutrient agar plate and then incubated at 37°C for 24 h. The starter's lowest concentration without bacterial growth was identified and taken as Minimum Bactericidal Concentration [25].

### 2.9. Determination of the Starters' Contents in Organic Acids, Sugar, and Volatile Compounds

Organic acids, sugar, and volatile compounds were determined using the high performance liquid chromatography (HPLC) method [26]. An amount (25 mg) of each fermented starter samples was mixed with 1 mL of 5 mM H_2_SO_4_ in screw-capped tubes. The mixtures were centrifuged (12000*g* for 10 min) and the supernatants filtered with 0.45 *μ*m filter. The separation of organic acids and the volatile compounds was achieved with an Aminex HPX-87H HPLC column (Bio-Rad Labs., Richmond, Calif., USA) at 45°C, using 5 mM H_2_SO_4_ as a mobile phase and the externally calibrated with standard solutions. The organic acids and volatile compound were identified and quantified by comparison of their retention times with these standard acids.

The content of a compound (mg/100 g of dry matter) = (SRe ×  *F*  × 10000)/(CRs × PE × MS); SRe is the response surface of the sugar in the sample; *F* is the dilution factor; CRs is the standard response factor; and PE: peak area/concentration (mg/mL).

### 2.10. Starters' Chemical Screening

Chemical screening of the “kpètè-kpètè” was carried out using the method of Houghton and Raman [27].

### 2.11. Data Analysis

Statistical analysis of antibacterial data was done with Excel 2010 and Graph Pad Prism 5. The chemical compounds data were analyzed using SAS (SAS Institute, Cary, NC, USA) to determine the significant difference between the various treatments. The mean difference was determined by Student Newman-Keuls test (*p* < 0.05). For normal distributions, the rate of organic acids, sugars, and volatile compound obtained was processed with 2 arcsin $\sqrt{n}$ [28], where *n* is the actual value.

## 3. Results

### 3.1. Inhibition of Reference Strains by the Starter “Kpètè-Kpètè” Tested

**Figure 1:**
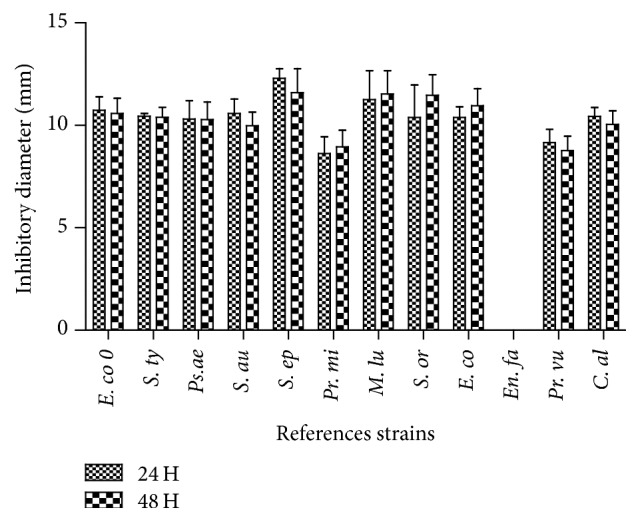
Medium inhibitory diameter of tchoukoutou's starter on reference strains after 24 h and 48 h.* E. co 0*:* Escherichia coli* O157,* S. ty*:* Salmonella typhimurium*,* Ps. ae*:* Pseudomonas aeruginos*a,* S. au*:* Staphylococcus aureus*,* S. ep*:* Staphylococcus epidermidis*,* Pr. mi*:* Proteus mirabilis*,* M. lu*:* Micrococcus luteus*,* S. or*:* Streptococcus oralis*,* E. co*:* Escherichia coli*,* En. fa*:* Enterococcus faecalis* ATCC 29212,* Pr. vu*:* Proteus vulgaris*,* C. al*:* Candida albicans*.

The collected “kpètè-kpètè” samples showed an antibacterial effect on some reference strains. The majority of the collected ferment in the four localities exhibited antibacterial effect on 91.67% (11/12) of the reference strains. The only one tested reference strain that was not inhibited by the starter was* Enterococcus faecalis*.  Figure 1 shows the compilation of inhibition diameter measured after 24 and 48 hours of incubation. Considering the sensitive strains, the mean inhibitory diameter zones vary from 8 mm (*Proteus mirabilis*) to 12.50 mm (*Staphylococcus epidermidis*). Our data shows that there was not a significant variation between diameter recorded at 24 h and those of 48 h (*p* > 0.05).

### 3.2. Antimicrobial Activity of “Kpètè-Kpètè” on Some Pathogenic Bacteria

**Figure 2:**
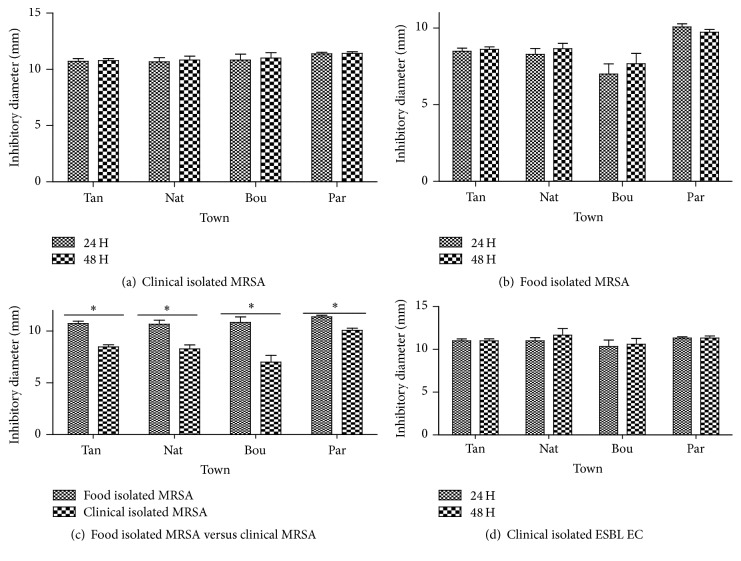
Mean inhibition zones' diameter of starter on some isolated pathogenic strains after incubation (24 and 48 h). Tan: Tanguiéta, Nat: Natitingou, Bou: Boukoumbé, Par: Parakou, MRSA: Methicillin Resistant* S. aureus*, and ESBL EC: extended-spectrum beta-lactamases* E. coli. ∗*: *p* < 0.05.

The antibacterial activities of “kpètè-kpètè” collected from four towns on some pathogenic food and clinical isolated bacteria are compiled on Figure 2. Thus, we observed that clinical isolated MRSA are sensitive to the tested “kpètè-kpètè” independently from their collection area (Figure 2(a)). This sensitivity was higher with the starter collected at Parakou (11.40 mm) and less with those from Tanguiéta (10.75 mm). However, considering the starter collection area, the recorded difference from a starter to another is not significant (*p* > 0.05). Our data shows that the food isolated MRSA are more sensitive to “kpètè-kpètè” collected at Parakou (9.89 mm) (Figure 2(b)). The recorded inhibition diameters of “kpètè-kpètè” on pathogenic bacteria did not vary over the incubation time. Globally, the clinical MRSA are more sensitive to “kpètè-kpètè” than food isolated MRSA (*p* < 0.05) (Figure 2(c)).

The clinical isolated extended-spectrum beta-lactamases* E. coli* tested strains are sensitive to “kpètè-kpètè” independently from their origin (Figure 2(d)). The inhibitory diameter zones of the starter tested vary slightly from the starters collected at Boukoumbé (10.50 mm) to those of Natitingou (12 mm) from a starter to another; but the difference is not statistically significant (*p* > 0.05). The recorded diameters did not vary over the time.

### 3.3. Antibacterial Activity of Lactic Acid Bacteria Isolates on Pathogenic Strains

**Figure 3:**
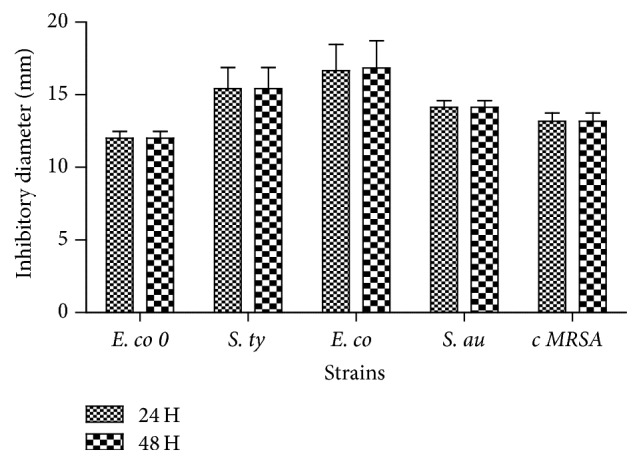
Lactic acid bacteria isolates' antimicrobial activity on some pathogenic strains after 24 h and 48 h of incubation. E. co 0:* E. coli* O157: H7 ATCC 700728, S. ty:* Salmonella typhi *R 30951401, E. co:* E. coli *ATCC 25922, S. au:* S. aureus *ATCC 29213, and c MRSA: clinical isolated MRSA.

The antibacterial activity of the lactic acid bacteria isolates showed that all pathogenic strains tested (6/6) are sensitive to the isolates independently from their collection area (Figure 3). It was observed that the inhibition diameters zones vary according not only to the pathogens strains but also to the lactic acid bacteria isolates. The results also showed that there was no significant difference between the isolates inhibition diameters in the time (*p* > 0.05).* E. coli* (16.75 mm) and* Salmonella typhi* are the most sensitive (15.43 mm) strains to lactic acid bacteria isolates. The lower diameters were obtained with clinical isolated MRSA strains (13.18 mm) and* E. coli O157 *strain (12 mm).

### 3.4. Sensitivity of Antagonistic Substances to Enzymes

The antibacterial compounds identified have no effect on three proteolytic enzymes (chymotrypsin, trypsin, and pepsin), indicating that the inhibitory compounds are protein, a general characteristic of bacteriocin (Table 1). There was not inhibition zone of the bacteriocin extracts in presence of various proteolytic enzymes. However, in the presence of amylase, lipase, and catalase we noticed a zone of inhibition.

### 3.5. Minimum Inhibitory and Bacterial Concentrations of Starter on Some References and Pathogenic Strains

The MIC and MBC vary depending on the types of strains and starter (Table 2). However, there was no significant difference between these different concentrations. So, the MIC varies from 0.3125 mg/mL (clinical MRSA with starter collected from Tanguiéta) to 5 mg/mL (Table 2), whereas the MBC varied from 1.25 mg/mL (*S. epidermidis* and clinical MRSA with the starter of Boukoumbé) to 20 mg/mL. These results showed that the starter can have bacteriostatic and bactericidal effect on reference strains and MRSA. Indeed, the ratio of the two parameters indicates bactericidal effect of the starters on references strains (*Pseudomonas aeruginosa, Salmonella typhi, E. coli ATCC 25922, S. epidermidis, Proteus mirabilis, Proteus vulgaris, Streptococcus oralis, *and* Candida albicans*) and clinical isolated MRSA (Table 2).

### 3.6. Effect of Lactic Acid Bacterial against* E. coli* ATCC25922

Table 3 shows inhibition diameters of lactic acid bacteria against* E. coli* ATCC 25922 strain. The different dilutions of supernatant obtained from lactic acid bacteria culture's showed inhibition diameters ranging from 6 mm to 17 mm up to a dilution factor of 256 above which no activity is observed. This dilution factor (256) represents thus the CMI of the assumed bacteriocin produced by lactic acid bacteria. However, it is noticed that the inhibition power decreases when the dilution of the supernatant increases.

### 3.7. Production of Organic Acid, Sugar, and Volatile Compounds in Starter of “*Tchoukoutou*”

Table 4 shows the concentration of organic acid, sugar, and volatile compounds in a starter of* tchoukoutou*. In this study, the organic acids produced and detected were lactic and propionic acids. Lactic acid was the main organic produced in the starter culture (*p* < 0.05). The lactic acid content in starter of* tchoukoutou* varied between 0.49 mg/100 g to 0.72 mg/100 g whereas the propionic acid content varied between 0.12 mg/100 g to 0.30 mg/100 g.

The principal volatile compound detected and identified in* tchoukoutou's* starter was ethanol. Thus, the level of this alcohol varied between 1.46 mg/100 g and 1.78 mg/100 g. There was a significant difference (*p* < 0.01) between the ethanol concentrations of starter of* tchoukoutou* from different regions.

The sugars detected in starter were raffinose, maltose, glucose, and fructose. Raffinose was the main sugar produced in starter with concentrations ranging from 0.27 to 0.35 mg/100 g and the fructose was the less produced (0.13–0.15 mg/100 g). There was no significant difference between raffinose and fructose levels (*p* > 0.05) but there was a significant difference (*p* < 0.01) in the levels of maltose and glucose in the starter.

### 3.8. Chemical Composition of the Tchoukoutou's Starter

The chemical screening of starter samples showed that the main chemical compounds were polyphenolic compounds (tannins, catechic tannins, flavonoids, anthocyanin, and leucoanthocyanes), mucilage, reducing compounds, triterpenoid, and steroids (Table 5). Other compounds such as alkaloids, quinone derivatives, saponosides, coumarin, and anthracene derivatives are absent in the starter.

## 4. Discussion

The “kpètè-kpètè” and the associated lactic acid bacteria have a remarkable antimicrobial potential on the majority of tested microorganisms (Figures 1–3). Thus, it appear that many pathogenic strains such as* Candida albicans, Salmonella typhi, E. coli*, and* S. aureus* were inhibited by the starter “kpètè-kpètè” (Figure 1). These results confirm those reported by Silva de et al. [29] in their study on antimicrobial activity of strains involved in the fermentation of “kefir.” This activity is very interesting and can explain the large inhibition proportion of spoilage germs responsible for poisoning during fermentation process. To support this, some lactic acid bacteria strains isolated from many African fermented foods are reported to produce antimicrobial substances against several spoilage microorganisms [30]. So, the inhibition effect may be due to the action of lactic bacteria contained in the tested starter. Indeed, several studies suggested that the antimicrobial effect of the lactic acid bacteria isolated from fermented paste of maize is mainly due to the effect of pH, H_2_O_2_, or bacteriocin [31].

Among the pathogenic microorganisms tested, only* Enterococcus faecalis *ATCC 29212 was resistant to the tested “kpètè-kpètè.” Meanwhile, this microorganism is reported to be sensitive to the lactic acid bacteria isolated in Burkina Faso from milk [21]. This difference may be explained by the fact that the active substance (bacteriocins) produced by lactic acid bacteria of the milk is not the same as those produced by lactic acid bacteria isolated from the “kpètè-kpètè.” In addition it could be also due to lactic acid bacteria strains implicated and their culture conditions because the antibacterial effect may vary depending not only on the lactic acid bacteria strain but also on the cultures conditions” [32].

Concerning the results of antimicrobial activities on* E. coli*,* E. coli* O157, and* S. aureus* recorded in this study, they are similar to those of by Savadogo et al. [21] in their study on antimicrobial activities of lactic acid bacteria strains isolated from Burkina Faso's fermented milk. Indeed, in their study, they show that lactic acid bacteria exhibited antimicrobial activity on pathogenic strains such as* S. aureus *and* E. coli*.

Both “kpètè-kpètè” and lactic acid bacteria showed a very interesting activity against methicillin resistant* S. aureus* isolated from food and clinical (Figures 2 and 3). Thus, the tested starter and lactic acid bacteria have an inhibitory effect on pathogenic bacteria as previously reported for bacteria isolated from traditional fermented Algerian milk [33]. The lactic acid bacteria are previously reported to be able to eliminate about 99% of multiresistant bacteria such as methicillin resistant* S. aureus *[34]. In addition, our data shows that the antimicrobial activity was completely inhibited by the proteolytic enzymes action (Table 1). So, the biologically active fraction of the inhibitory substance may have a protein nature. Though the nature of the bacteriocin is very important to determine, it is more interesting to confirm these results in vitro as done by other authors [35, 36].

According to our data, the in vitro test shows the smallest minimum inhibitory concentration (0.31 mg/mL) with the methicillin resistant* S. aureus *strains in comparison to the value (0.625 mg/mL) of some reference strains (*Pseudomonas aeruginosa*,* S. aureus*, and* S. epidermidis*) (Table 2). Thus the “kpètè-kpètè” can have an inhibiting effect at low dose on the pathogenic strains such as methicillin resistant* S. aureus*.

The ratio between the CMB and CMI shows that the starter can have bactericidal or bacteriostatic effect depending on the tested strains (Table 2). However, there is less bactericidal effect with the reference strains (*Pseudomonas aeruginosa*,* Salmonella typhi*,* E. coli ATCC 25922*,* S. epidermidis*,* Proteus vulgaris*,* Streptococcus oralis*, and* Candida albicans*) and clinical isolated methicillin resistant* S. aureus*. These results are not similar to those found by Dramane et al. [37] when they showed that the extract of* Erythrina senegalensis *has bactericidal effect on several pathogenic references strains (*Candida albicans*,* Enterococcus faecalis*,* S. epidermidis*,* S. aureus*,* Proteus mirabilis*,* E. coli*, and* Pseudomonas aeruginosa*). The difference of the bactericidal capacity could be explained not only by the fact that the authors used plants extracts instead of a fermented food but also by the fact that the active substance of each product acts differently on the strains.

The analysis of the starter's composition by HPLC revealed the presence of the organic acids in particular the lactic and acetic acid (Table 4). These compounds may then be mainly responsible for the kpètè-kpètè's antimicrobial activity. Indeed, the organic acids were indexed in the antimicrobial activity of lactic acid bacteria isolates from Ghanaian fermented maize called* “*kenkey*” *[38]. In addition, the chemical screening of the starter showed that it contains several compounds such as steroids, polyphenolic compounds (tannins, flavonoids, and anthocyans), mucilage, and reducing compounds (Table 5). The presence of tannins and flavonoids in the starter suggests that those “kpètè-kpètè” may have some biological and pharmaceutical (antidiarrheic, antibacterial, antiviral, and anticarcinogenic) properties [39, 40]. So these compounds in the starter can explain its antimicrobial activity in addition to the organic acid effect. Indeed, these secondary metabolites have been recognized as the base of medicinal plants' antibacterial properties [41–44]. The results of this work give us the probable origin of good antimicrobial activity of the starter and lactic acid bacteria observed in this study. Apart from the antibacterial effect of those starters, the presence of polyphenolic compounds indicates that it may have other biological activities such as antioxidant activity.

## 5. Conclusion

Tchoukoutou starter named “kpètè-kpètè” prepared in Benin displayed antimicrobial activity. These properties are expressed not only by the production of the bacteriocin (protein substance), but also by the chemical compounds contained in the starters. Thus, the lactic acid bacteria present in the starters are able to produce lactic acid, acetic acid, ethanol, peptides (bacteriocins), and other biologically active compounds which increase the starter capacity to inhibit or kill pathogenic strains. So, these results provide a scientific support to the traditional use of the “kpètè-kpètè” in treatment of bacterial infections such as diarrhea and dysentery. The “kpètè-kpètè” can therefore be used for the elaboration of a probiotic food ingredient for human and animals in order to effectively control resistant microorganisms. This starter can be also used to control food and clinical multiresistant bacteria.

## Figures and Tables

**Table 1:** The inhibitory diameters recorded on bacterial culture after enzymatic hydrolysis.

LAB strain	Reference strains	Chymotrypsin	Trypsin	Pepsin	Lipase	Amylase	Catalase
LAB 1	*Candida albicans*	0 mm	0 mm	0 mm	8 mm	8 mm	10 mm
LAB 1	*Enterococcus faecalis*	0 mm	0 mm	0 mm	9 mm	9 mm	8 mm
LAB 2	*Escherichia coli* O 157	0 mm	0 mm	0 mm	6 mm	5 mm	9 mm
LAB 2	*Escherichia coli*	0 mm	0 mm	0 mm	7 mm	8 mm	9 mm
LAB 3	*Micrococcus luteus*	0 mm	0 mm	0 mm	0 mm	0 mm	11 mm
LAB 4	*Proteus mirabilis*	0 mm	0 mm	0 mm	8 mm	6 mm	10 mm
LAB 6	*Proteus vulgaris*	0 mm	0 mm	0 mm	7 mm	0 mm	10 mm
LAB 7	*Pseudomonas aeruginosa*	0 mm	0 mm	0 mm	8 mm	7 mm	10 mm
LAB 8	*Salmonella typhimurium*	0 mm	0 mm	0 mm	6 mm	5 mm	7 mm
LAB 10	*Staphylococcus aureus*	0 mm	0 mm	0 mm	8 mm	7 mm	9 mm
LAB 10	*S. epidermidis*	0 mm	0 mm	0 mm	6 mm	6 mm	8 mm
LAB 10	*Streptococcus oralis*	0 mm	0 mm	0 mm	6 mm	0 mm	9 mm

**Table 2:** Compilation of the tested starters' minimum inhibitory and bactericidal concentration on some references strains.

Pathogenic strains	Tanguiéta			Natitingou			Boukoumbé			Parakou		
	MIC (mg/mL)	MBC (mg/m)	MBC/MIC	MIC (mg/mL)	MBC (mg/m)	MBC/MIC	MIC (mg/mL)	MBC (mg/m)	MBC/MIC	MIC (mg/mL)	MBC (mg/mL)	MBC/MIC
*Escherichia coli *O157	1.25	*C* > 20	—	5	*C* > 20	—	2.5	10	4	2.5	10	4
*Pseudomonas aeruginosa*	0.62	10	16	5	*C* > 20	—	2.5	5	2^**∗**^	2.5	10	4
*Salmonella typhi*	1.25	20	16	5	5	1^**∗**^	5	10	2^**∗**^	2.5	20	8
*Staphylococcus aureus*	0.62	20	32	5	*C* > 20	—	2.5	*C* > 20	—	1.25	20	16
*Proteus mirabilis*	2.5	10	4	5	*C* > 20	—	1.25	5	4	1.25	2.5	2^**∗**^
*E. coli *ATCC 25922	2.5	5	2^**∗**^	5	20	4	2.5	10	4	1.25	10	8
*S. epidermidis*	0.62	**1.25**	2^**∗**^	5	*C* > 20	—	2.5	5	2^**∗**^	2.5	10	4
*Proteus vulgaris*	1.25	5	4	5	10	2^**∗**^	2.5	5	2^**∗**^	2.5	20	8
*Streptococcus oralis*	1.25	5	4	5	*C* > 20	—	5	10	2^**∗**^	2.5	*C* > 20	—
*Candida albicans*	2.5	5	2^**∗**^	1.25	20	16	2.5	5	2^**∗**^	2.5	20	8
*ESBL E. coli*	1.25	5	4	2.5	20	8	2.5	20	8	2.5	5	2^**∗**^
Clinical isolated MRSA	2.5	5	2^**∗**^	2.5	10	4	0.312	5	16	0.62	**1.25**	2^**∗**^
Food isolated MRSA	1.25	5	4	1.25	**5**	4	1.25	**5**	4	0.62	**2.5**	4

MRSA: methicillin resistance *S. aureus*, ESBL EC: extended-spectrum beta-lactamases* E. coli,* MIC: minimum inhibitory concentration, MBC: minimum bactericidal concentration, *with*  *∗*: bactericidal effects, and *without*  *∗*: bacteriostatic effects.

**Table 3:** Effect of lactic acid bacterial supernatant dilution against *E. coli* ATCC25922.

	Dilutions									
	1	2	4	8	16	32	64	128	256	512
Inhibition diameter (mm)	17	17	15	15	12	11	10	8	6	0

**Table 4:** Organic acids, sugars contents and volatile compound (mg/100 g) of the traditional beer tchoukoutou's starter (mean ± standard error).

Commune	Raffinose	Maltose	Glucose	Fructose	Lactic acid	Propionic acid	Acetic acid	Ethanol
Boukoumbé	0.27 ± 0.02^a*∗*^	0.23 ± 0.01^a^	0.22 ± 0.03^a^	0.14 ± 0.01^a^	0.49 ± 0.03^c^	0.12 ± 0.01^c^	0.33 ± 0.11^b^	1.46 ± 0.08^b^
Natitingou	0.35 ± 0.04^a^	0.25 ± 0.01^a^	0.16 ± 0.03^b^	0.15 ± 0.01^a^	0.63 ± 0.04^ba^	0.20 ± 0.02^b^	0.63 ± 0.04^ba^	2.06 ± 0.15^a^
Parakou	0.25 ± 0.03^a^	0.16 ± 0.02^b^	0.13 ± 0.01^b^	0.14 ± 0.02^a^	0.63 ± 0.04^bc^	0.63 ± 0.04^ba^	0.63 ± 0.04^ba^	1.35 ± 0.14^b^
Tanguiéta	0.35 ± 0.04^a^	0.24 ± 0.01^a^	0.10 ± 0.01^b^	0.13 ± 0.01^a^	0.72 ± 0.03^a^	0.30 ± 0.14^a^	0.94 ± 0.12^a^	0.63 ± 0.04^ba^
Probabilité	0.06	0.0002	0.0023	0.84	0.0007	<0.0001	0.008	0.001

^*∗*^Data with the same letters are not significantly different (*p* > 0.05).

**Table 5:** Chemical content of the tchoukoutou's starter.

Chemical groups	Subgroups	Test results
Alkaloids		−

*Polyphenolic compound*	Tannins	++
	Catechic tannins	++
	Gallic tannins	−
	Flavonoids	++ flavone
	Anthocyanin	−
	Leucoanthocyanes	++

Quinone derivatives		−

Saponosides		−

Triterpenoids		++
Steroids		++
Cardenolides		−

Cyanogenic compound		−

Mucilage		+++

Coumarins		−

Reducing compound		+++

Anthracene derivatives	Free anthracene	−
	Combine anthracene:	
	-O-Glycosides	−
	-C-Glycosides	−

+++: great presence; ++: fair presence; −: absence.
